# S-1-based concurrent chemoradiotherapy plus nimotuzumab in patients with locally advanced esophageal squamous cell carcinoma who failed neoadjuvant therapy: a real-world prospective study

**DOI:** 10.1080/15384047.2024.2417464

**Published:** 2024-10-27

**Authors:** Xin Wang, Guojie Feng, Xiongtao Yang, Nuo Yu, Ziyu Zheng, Jiao Li, Xiaozheng Kang, Xiankai Chen, Ruixiang Zhang, Yong Li, Zhen Wang, Lei Deng, Tao Zhang, Wenyang Liu, Jianyang Wang, Wenqing Wang, Qinfu Feng, Zefen Xiao, Zongmei Zhou, Nan Bi, Yin Li, Jianjun Qin

**Affiliations:** a Department of Radiation Oncology, National Cancer Center/National Clinical Research Center for Cancer/Cancer Hospital, Chinese Academy of Medical Sciences & Peking Union Medical College, Beijing, China; b Department of Oncology, Beijing Changping Hospital, Beijing, China; c Department of Thoracic Surgery, National Cancer Center/National Clinical Research Center for Cancer/Cancer Hospital, Chinese Academy of Medical Sciences & Peking Union Medical College, Beijing, China

**Keywords:** Concurrent chemoradiotherapy, S-1, nimotuzumab, locally advanced ESCC, real-world study, chemoimmunotherapy

## Abstract

**Purpose:**

This prospective study in a real-world setting investigated the feasibility and safety of S-1 plus nimotuzumab (S-1-Nimo) based concurrent chemoradiotherapy (CCRT) in locally advanced esophageal squamous cell carcinoma (LA-ESCC) patients who failed to neoadjuvant chemotherapy or chemoimmunotherapy.

**Methods:**

LA-ESCC patients who failed to converse to resectable disease after neoadjuvant chemotherapy or chemoimmunotherapy were enrolled to receive the 4-week S-1-Nimo regimen of radiotherapy (40 Gy in 20 fractions, 5 days per week), S-1 chemotherapy, and nimotuzumab. Then, after surgical assessments, patients evaluated as resectable disease received surgery; patients with unresectable disease continued to receive definitive radiotherapy (50–60 Gy in 25–30 fractions, 5 days per week) concurrently with S-1-Nimo. The primary endpoint was event-free survival (EFS).

**Results:**

Sixty-four patients were enrolled and evaluated. The median follow-up time was 23.2 months. Median EFS was 9.6 (95% confidence interval [CI], 7.1–14.0) months, with an estimated 2-year EFS rate of 24.2%. The median overall survival (OS) and the estimated OS rate at 2 years were 13.4 (95% CI, 10.3–17.5) months and 31.2%, respectively. Twelve underwent surgery, with a surgical conversion rate of 18.8% and an R0 resection rate of 100.0%. Subgroup analysis identified the significantly prolonged EFS and OS in patients who experienced radical surgery (median EFS, not reached vs. 8.7 months; *p* = .0117. median OS, 24.9 vs. 10.6 months; *p* = .0205) as compared to those treated with CCRT. Of 64 patients, grade 3 adverse events mainly included radiation esophagitis (4.7%), anemia (1.6%), and thrombocytopenia (1.6%).

**Conclusion:**

The study demonstrated the reasonable efficacy and promising safety of the S-1-Nimo-based CCRT in LA-ESCC patients with failure to neoadjuvant chemotherapy or chemoimmunotherapy.

## Background

Esophageal cancer, ranking seventh most common malignancy worldwide, is histologically classified as esophageal adenocarcinoma (EAC) and esophageal squamous cell carcinoma (ESCC).^1^ Compared to EAC being more prevalent in Europe and America, ESCC accounts for approximately 90% of global esophageal cancer cases and exhibits the highest incidences in eastern Asia (especially in China).^2^ The KEYNOTE-181 and CHECKMATE-577 trials showed that ESCC responded better to immunotherapy than EAC;^3–5^ thus, neoadjuvant chemoimmunotherapy is widely explored in China beyond standard neoadjuvant chemoradiotherapy.^6,7^ Results from numerous phase II studies (TD-NICE, NICE, NIC-ESCC2019, etc.) also indicated the feasibility of neoadjuvant chemoimmunotherapy, with a pathological complete response (pCR) rate of 25%–42.5% and objective response rate (ORR, 46.7%–66.7%).^8–10^Besides, several additional phase III randomized controlled trials are currently underway to further validate the role of neoadjuvant chemoimmunotherapy in locally advanced ESCC (LA-ESCC).^11,12^ ESCORT-NEO randomized trial demonstrated that neoadjuvant camrelizumab with chemotherapy significantly improved (pCR, 15.4%–28.0% vs. 4.7%; odds ratio, 3.81–8.11) over neoadjuvant chemotherapy alone in LA-ESCC.^13^ As such, neoadjuvant chemoimmunotherapy has been recommended in the 2024 Chinese Society of Clinical Oncology (CSCO) guidelines.

However, some patients did not benefit from chemoimmunotherapy or eventually progressed or relapsed due to the absence of reliable predictive biomarkers or acquired resistance.^14,15^ As demonstrated in the KEYNOTE-590 trial with pembrolizumab plus chemotherapy, there was no significant survival improvement for 32% of patients with low programmed death ligand 1 (PD-L1) expression.^14^ Besides, the 2-year outcomes of the NICE study of neoadjuvant camrelizumab plus chemotherapy showed that 37.3% of patients developed disease recurrence and 2-year overall survival (OS) and recurrence-free survival rates were, respectively, 78.1% and 67.9%.^15^ However, in reference to second-line treatments following the failure of first-line treatment, the response rates typically do not exceed ~20%.^16^ As such, we hypothesize that among these patients who failed to chemoimmunotherapy, the radiation-based combination represents one promising later-line treatment option. Usually, concurrent chemoradiotherapy (CCRT) is the preferred treatment, with one commonly used chemotherapy regimen being paclitaxel and platinum. Nonetheless, patients with previous exposure to 2–4 cycles of chemotherapy typically exhibit poor tolerance (particularly hematological toxicity) to CCRT, making it challenging to sustain continuous exposure to paclitaxel and platinum. Thus, consideration should be given to switching to a less toxic and more active treatment regimen.

Oral S-1 (tegafur, gimeracil, and oteracil) chemotherapy was widely used in East Asia and exhibited reduced toxicity, enhanced radiosensitivity, and improved survival. As evidenced by our prior data on patients aged ≥70 years, S-1-based CCRT showed a median OS of 28.1 months with an 8-month prolongation and favorable safety versus radiotherapy alone.^17^ In parallel, the cooperation of anti-epidermal growth factor receptor nimotuzumab and CCRT has demonstrated an improved complete response rate and ORR in the ESCC treatment.^18^ Our recent work has also shown the promising efficacy and safety of combining nimotuzumab with S-1 chemotherapy and concurrent radiotherapy in malnourished and elderly patients with locally advanced EC.^19^ Together, we hypothesize that the combination of the S-1-based CCRT and nimotuzumab (defined as the S-1-Nimo regimen) may reduce toxicity while enhancing antitumor efficacy in patients with failure to neoadjuvant therapy; nonetheless, no data is available.

To establish the groundwork for a forthcoming randomized clinical trial aimed at confirming the clinical efficacy of the S-1-Nimo regimen, we initiated a real-world study to assess the safety and feasibility of administering this combination in a select group of LA-ESCC patients who failed the neoadjuvant therapy.

## Materials and methods

### Study design

This prospective study in a real-world setting (Trial registration ID: NCT04821778) was conducted at the Chinese Academy of Medical Sciences. It was approved by the Ethics Committee of the Chinese Academy of Medical Sciences (No. 21/089-2740). The study was conducted in accordance with the International Conference on Harmonization of Good Clinical Practice guidelines, the Declaration of Helsinki, and applicable local laws and regulations. Written informed consent was obtained from each patient.

### Patient population

Eligible patients aged ≥18 years with histologically confirmed stage III-IVb (IVb limited to lymph-node metastasis in the supraclavicular area) ESCC who failed to converse to resectable disease after neoadjuvant chemotherapy or chemoimmunotherapy were enrolled from July 2020 to July 2022. The neoadjuvant therapy failure was defined as being unresponsive (stable disease [SD]/locoregional progressive disease [PD]) to treatment or being unresectable as evaluated by surgeons after neoadjuvant therapy. Tumor staging was examined before neoadjuvant therapy according to the 8th TNM staging system of the American Joint Committee on Cancer (AJCC). Other inclusion criteria were Eastern Cooperative Oncology Group performance status (ECOG PS) of ≤2, and normal organ and marrow function. Patients with other malignancies (other than cured cervical carcinoma in situ or non-melanoma skin cancer) within 5 years and participation in other clinical trials currently or within 4 weeks prior to the study were ineligible for this study.

### Treatment

All patients underwent computed tomography (CT) simulation in the supine position, with CT images obtained at 5 mm thickness throughout the entire neck, thorax, and upper abdomen. Patients were treated with 4-week S-1-Nimo regimen comprising radiotherapy at a dose of 40 Gy in 20 fractions for 5 days weekly in volumetric-modulated arc therapy (VMAT), concurrent 40–60 mg/m^2^ body surface area of oral S-1 twice daily, and 400 mg of intravenous nimotuzumab once a week. After the completion of the 4-week S-1-Nimo regimen, the surgeons assessed the patient eligibility for potentially curative surgery based on the results of enhanced CT. Minimally invasive or open McKeown esophagectomy combined with a two- or three-field lymphadenectomy (only if metastatic supraclavicular lymph nodes were presented) was performed on resectable tumors. Inoperable patients could continue a 1–2-week combination of radiotherapy at 50–60 Gy in 25–30 fractions for 5 days per week, concurrent S-1 (40–60 mg/m^2^ body surface area twice daily), and nimotuzumab (400 mg once a week). Gross target volume (GTV) included a primary tumor and the affected lymph nodes identified through imaging. Clinical target volume (CTV) comprised CTVt (GTV for the primary tumor plus a 3 cm margin craniocaudally and 0.5 cm margin laterally) and CTVnd (GTV for lymph nodes plus a 0.5–1.0 cm radial margin). The planning target volume was created by adding a 0.5 cm radial margin to the CTV. The lungs, heart, esophagus, spinal cord, stomach, bowel, and kidneys were delineated as organs at risk for dose constraints.

S-1 and nimotuzumab were discontinued when grade 3 or higher drug-related toxicity occurred. The dose reductions of S-1 and nimotuzumab were not considered.

### Assessments and outcomes

Following surgery, resection specimens underwent pathological examination to assess resection margin status and tumor regression grade (TRG). R0 resection was defined as the absence of cancer cells at resection margins. TRG was classified according to the Mandard-TRG system: TRG1 (no residual cancer cells), which was equivalent to a pCR; TRG2 (scattered residual tumor cells with massive fibrosis); TRG3 (coexistence of fibrosis and residual tumor, but predominant fibrosis); TRG4 (the presence of residual tumor cells which outgrow the fibrosis); TRG5 (no evidence of regression).

The primary endpoint was event-free survival (EFS), defined as the duration from enrollment to the initial progression (locoregional tumor recurrence or distant metastasis) or any-cause death. Secondary endpoints were OS (the period from enrollment to all-caused death), surgical conversion rate, pCR rate (the proportion of patients with TRG1 in surgery group), major pathological response (MPR) rate (the proportion of patients with TRG1 and TRG2 in surgery group), R0 resection rate in surgery group, and safety (including adverse events [AEs] and postoperative complications). AEs were graded by the National Cancer Institute’s Common Toxicity Criteria for Adverse Events (NCI-CTCAE), version 5.0. Postoperative complications were described based on the Clavien – Dindo classification.

Follow-up assessments included clinical examination, biochemical tests, and cervical/chest/abdominal CT scan. Patients were followed-up once every 3 months for the first 2 years and every 6 months thereafter.

### Statistical analysis

Analysis of efficacy was mainly conducted in the full analysis set (FAS), defined as enrolled patients who had received the study treatment, regardless of whether they underwent surgery. Safety was analyzed in the safety analysis set (SS), which comprised the FAS population with safety records.

Categorical variables were summarized using frequencies (percentage [%]), and continuous variables were described by medians accompanied by interquartile ranges (IQR) or ranges. EFS and OS were assessed using the Kaplan–Meier method with a 95% confidence interval (CI) and compared using the log-rank test for between-group differences. Median follow-up was calculated by the reverse Kaplan–Meier method. Patients who were lost to follow-up or remained alive were censored in the final analysis. Using the Cox proportional hazards model to identify independent prognostic survival factors throughout univariate and multivariate analysis. The statistical analyses were performed using SAS v9.4 (SAS Institute, Cary, NC, USA). Significance was set at *p* < .05 for all two-sided tests conducted.

## Results

### Patient characteristics

**Figure 1.**
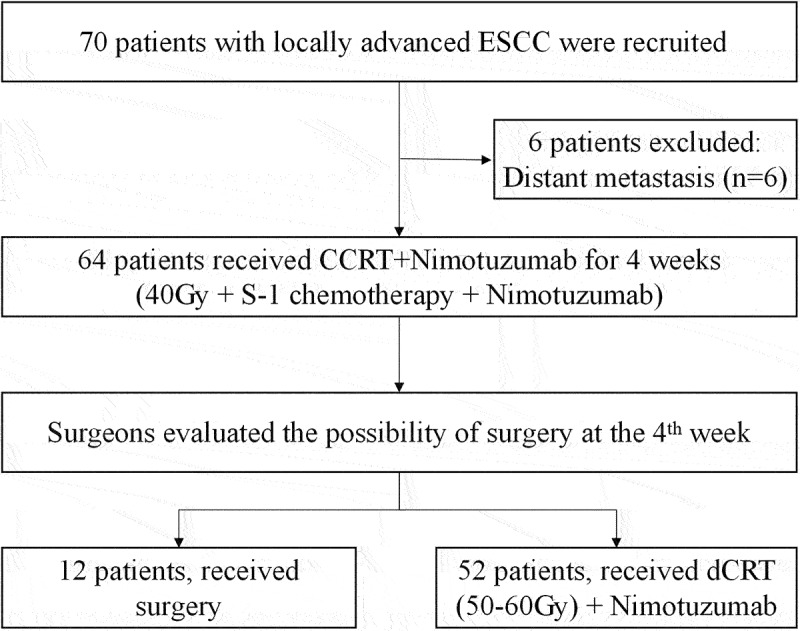
CONSORT diagram.

**Abbreviations**: ESCC, esophageal squamous cell carcinoma; dCRT, definitive CCRT.

From July 2020 to July 2022, 70 patients were screened and 6 were excluded due to distant metastasis (*n* = 6); 64 patients were enrolled and were included in the FAS and SS. Figure 1 showed the CONSORT diagram. The baseline characteristics of 64 patients were listed in Table 1. The median age was 65 (range, 49–76) years and most of the patients were male (59/64, 92.2%). Most patients had clinical stage IV disease (50/64, 78.1%). All cases received previous albumin-bound paclitaxel/platinum-based neoadjuvant therapy every three weeks (median cycle 3, range [2–8]), with chemotherapy plus programmed cell death protein 1 inhibitors (43/64, 67.1%) being common. Besides, 33 (51.6%) patients achieved PR after initial drug therapy but still were assessed as unresectable or difficult to receive radical surgery.

**Table 1.** Baseline characteristics.

Characteristics	FAS population (n = 64)
Age, years-median (range)	65 (49-76)
≥65	33 (61.6%)
≥70	11 (17.2%)
Male sex	59 (92.2%)
BMI, median (range)	21.9 (15.2-41.1)
Thin (BMI under 18.5)	9 (14.1%)
Normal (BMI 18.5-25.0)	38 (59.4%)
Overweight (BMI greater than 25.0 and under 30.0)	15 (23.4%)
Obese (BMI greater than 30.0)	2 (3.1%)
Smoking history	55 (85.9%)
Alcohol history	52 (81.3%)
Tumor length, cm-median (range)	5.8 (2.0-11.0)
>5 cm	38 (59.4%)
≤5 cm	26 (40.6%)
Primary tumor location	
Upper thoracic	17 (26.6%)
Middle thoracic	26 (40.6%)
Lower thoracic	21 (32.8%)
Prior neoadjuvant therapy	
Albumin-bound paclitaxel+platinum+anti-PD-1	43 (67.1%)
Albumin-bound paclitaxel+platinum-based chemotherapy	21 (32.8%)
Cycle number of neoadjuvant therapy, median (range)	3 (2-8)
<4	36 (56.3%)
≥4	28 (43.8%)
Specific treatment of neoadjuvant therapy	
21-day cycle	64 (100.0%)
T stage	
T2	3 (4.7%)
T3	28 (43.8%)
T4a	12 (18.8%)
T4b	21 (32.8%)
N stage	
N1	7 (10.9%)
N2	27 (42.2%)
N3	30 (46.9%)
Clinical tumor, node, metastases (TNM) stage	
III	14 (21.9%)
IVA	31 (48.4%)
IVB	19 (29.7%)
ECOG PS	
0	2 (3.1%)
1	57 (89.1%)
2	5 (7.8%)
Best response at neoadjuvant therapy stage	
PR	33 (51.6%)
SD	22 (34.4%)
PD	9 (14.1%)

Data were presented as the median (range) or n (%).

**Abbreviations**: FAS, full analysis set; BMI, body mass index; PD-1, programmed cell death protein 1; ECOG PS, Eastern Cooperative Oncology Group Performance Score; PR, partial response; SD, stable disease; PD, progressive disease.

### Treatment compliance

Eight (12.5%) patients did not complete concurrent chemotherapy due to anemia (grade 2, *n* = 3; grade 3, *n* = 1), grade 2 thrombocytopenia (*n* = 1), grade 3 nausea and vomiting (*n* = 1), grade 2 radiation esophagitis (*n* = 1), and grade 2 hyperbilirubinemia (*n* = 1). The median dose of S-1 chemotherapy was 2760 (IQR, 2100–3000) mg. Three (4.7%) patients did not complete radiotherapy because of grade 2 pneumonitis; the final radiation doses for these 3 patients were, respectively, 44, 48, and 39.6 Gy. Median PTV dose was 50.0 (range, 40.0–60.0) Gy. Besides, 5 (7.8%), 19 (29.7%), 23 (35.9%), and 14 (21.9%) patients completed 3, 4, 5, and 6 cycles of nimotuzumab, respectively.

Of all 64 patients, 12 proceeded with surgery resection with a successful conversion rate of 18.8%. The median number of positive lymph nodes was 0 (range, 0–16); no blood vessel invasion occurred in 12 patients. The mean number of lymph nodes resected was 37 (range, 26–48).

### Efficacy

**Figure 2.**
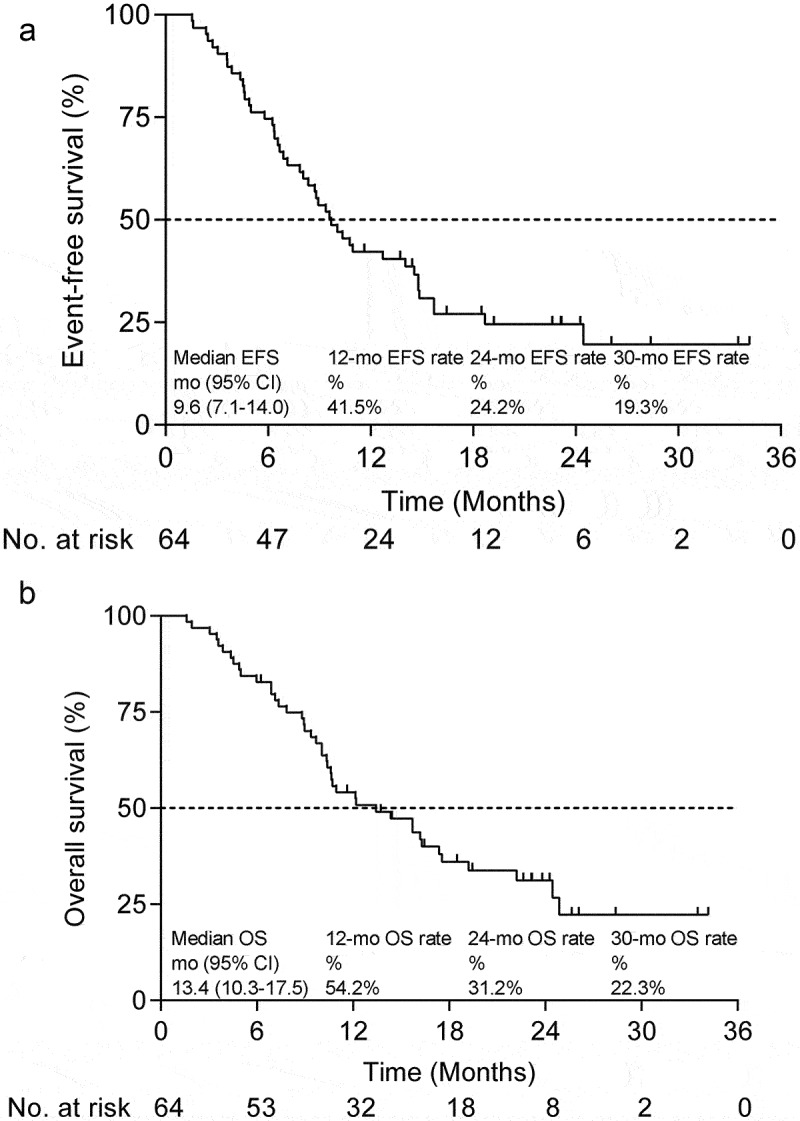
Efficacy outcomes.

As of 7 November 2023, the median follow-up was 23.2 months. The median EFS was 9.6 (95% CI, 7.1–14.0) months (Figure 2a); the estimated 1-year and 2-year EFS rates were, respectively, 41.5% and 24.2%. The median OS was 13.4 (95% CI, 10.3–17.5) months, with estimated 1-year and 2-year OS rates of 54.2% and 31.2%, respectively (Figure 2b).

(a) Kaplan–Meier curves for event-free survival in the FAS population (*n* = 64). (b) Kaplan–Meier curves for the overall survival of the FAS population (*n* = 64).

**Abbreviations**: FAS, full analysis set; OS, overall survival; EFS, event-free survival; mo, months; CI, confidence interval.

**Figure 3.**
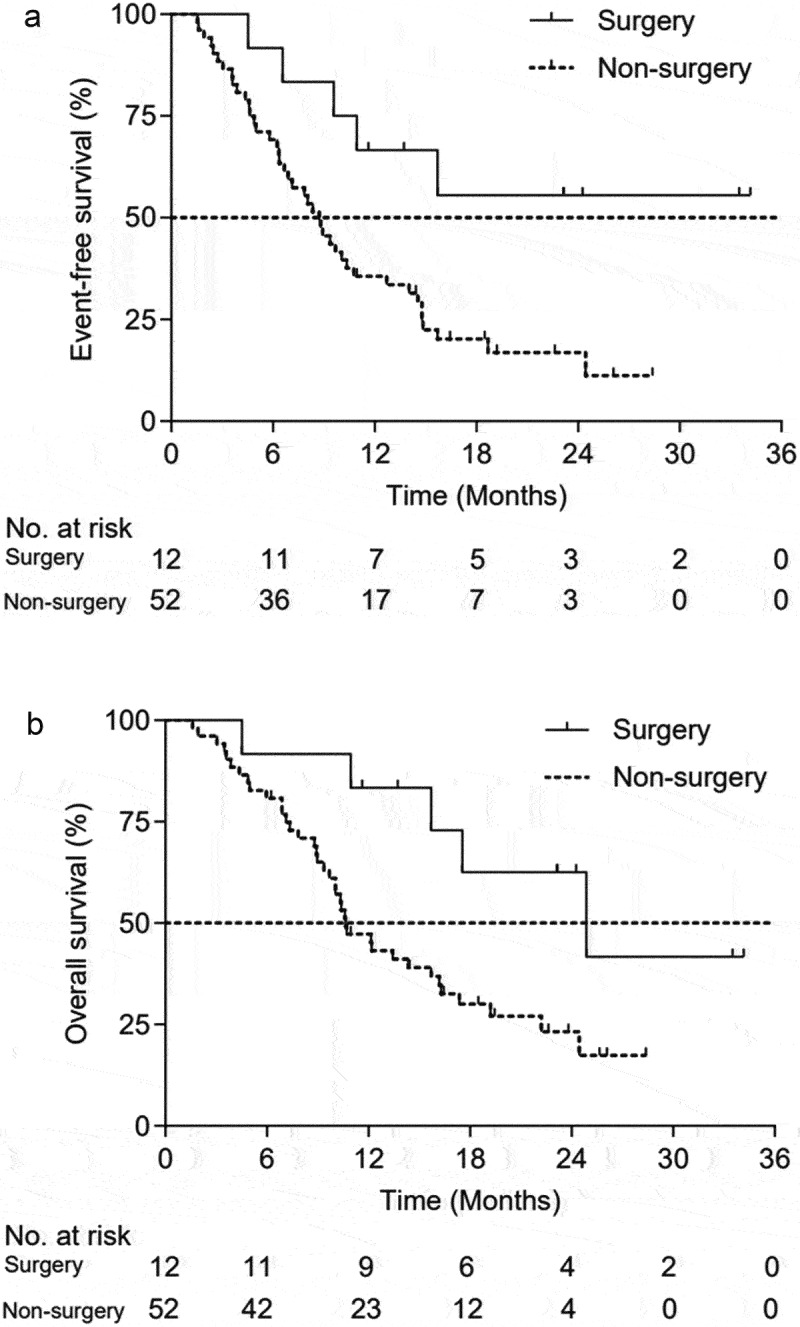
Efficacy outcomes grouped by surgery and non-surgery. (a) event-free survival, (b) overall survival.

To assess the consistency of treatment effect on OS and EFS, subgroup analyses were shown in Table S1. No significant association was identified across subgroups except for with or without surgery, tumor length, GTV volume. Median EFS (not reached vs. 8.7 months; *p* = .0117) and median OS (24.9 vs. 10.6 months; *p* = .0205) were significantly longer in the surgery group than those in the non-surgery group. The 1-year/2-year EFS rates in the surgical group were higher than those in the non-surgical group (66.67% vs. 35.63% at 1 year, 55.56% vs. 16.84% at 2 years), respectively (Figure 3a). The 1-year/2-year OS rates in the surgical group were also higher than those in the non-surgical group (83.33% vs. 47.28% at 1 year, 62.50% vs. 23.18% at 2 years), respectively (Figure 3b).

Moreover, Favorable EFS (14.8 vs. 7.1 months; *p* = .0100) and OS (22.2 vs. 10.4 months; *p* = .0060) also occurred in the patients who had baseline tumor length of ≤5 cm compared with those who had >5 cm. Compared to those with a GTV of >41 cm^3^, patients with a GTV of ≤41 (median) cm^3^ had better EFS (6.8 vs. 15.7 months; *p* < .0001) and OS (10.1 vs. 24.4 months; *p* < .0001). No significant difference of survival outcomes was observed between patients achieved PR or not after initial chemotherapy or chemoimmunotherapy.

Of the 12 patients who underwent surgery, the pCR (TRG1) rate and R0 resection rate were respectively 33.3% and 100.0%. TRG2, TRG3, and TRG4 were observed in 3 (25.0%), 4 (33.3%), and 1 (8.3%) patients, respectively. The MPR (TRG1/2) rate was 58.3%.

Among these 12 patients with surgery, 1 (8.3%) patient experienced regional recurrence after surgery, with recurrence times of 5.7 months; 2 (16.7%) cases experienced distant brain and lung metastasis, with metastatic times of 4.1 and 7.0 months, respectively. Of 52 non-surgery patients, 18 (34.6%) patients experienced regional recurrence (median recurrence time, 8.2 [range, 2.4–18.7] months); 4 (7.7%) patients, respectively, experienced distant lung, liver, bone, and lymph node metastasis (median recurrence time, 6.2 [range, 1.5–14.9] months).

### Safety and tolerability

Of 64 patients in the SAS, 60 (93.8%) patients experienced at least one AE (Table 2). Any-grade AEs mainly included radiation esophagitis (76.6%), leukopenia (34.4%), anemia (29.7%), fatigue (26.6%), and nausea (21.9%). Grade 3 AEs occurred in 3 (4.7%) patients, including radiation esophagitis (4.7%), anemia (1.6%), and thrombocytopenia (1.6%). No grade 4 or 5 or serious AEs occurred.

**Table 2.** Adverse events (AEs) in the SAS (*n* = 64).

	Safety population (n = 64)		
	Any grade	Grade 1-2	Grade 3
**Any events**	**60 (93.8%)**	**57 (89.1%)**	**3 (4.7%)**
Radiation esophagitis	49 (76.6%)	46 (71.9%)	3 (4.7%)
Leukopenia	22 (34.4%)	22 (34.4%)	0
Anemia	19 (29.7%)	18 (28.1%)	1 (1.6%)
Fatigue	17 (26.6%)	17 (26.6%)	0
Nausea	14 (21.9%)	14 (21.9%)	0
Anorexia	12 (18.8%)	12 (18.8%)	0
Neutropenia	12 (18.8%)	12 (18.8%)	0
Cough	11 (17.2%)	11 (17.2%)	0
Thrombocytopenia	5 (7.8%)	4 (6.3%)	1 (1.6%)
Vomiting	5 (7.8%)	5 (7.8%)	0
Fever	2 (3.1%)	2 (3.1%)	0
Radiation pneumonitis	1 (1.6%)	1 (1.6%)	0
Radiodermatitis	1 (1.6%)	1 (1.6%)	0
Blood bilirubin increased	1 (1.6%)	1 (1.6%)	0
Abnormal blood urea nitrogen (BUN)/creatinine (CRE)	1 (1.6%)	1 (1.6%)	0

Data were presented as n (%).

**Abbreviations**: SAS, safety analysis set.

The postoperative complications included fever (5/12, 41.7%), pleural effusion (5/12, 41.7%), subcutaneous emphysema of the chest wall (4/12, 33.3%), pneumothorax (2/12, 16.7%), and anastomotic fistula (1/12, 8.3%).

Two (3.1%) and six (9.4%) patients, respectively, required discontinuation of nimotuzumab and S-1 chemotherapy. Totally 44 (68.8%) deaths occurred; of whom, 38 (86.4%) died from tumor progression.

## Discussion

As far as we know, this is the first study that demonstrated the efficacy of S-1-Nimo-based radiotherapy for LA-ESCC patients who did not respond to prior chemo(immuno)therapy, with an encouraging EFS of 9.6 months and OS of 13.4 months and manageable safety profiles.

Several studies have shown promising efficacy with preoperative albumin-bound paclitaxel-based (radio)chemotherapy in LA-ESCC patients.^20,21^ Zhang et al. reported significant tumor downstage and encouraging pathological outcomes (pCR, 37.5%; MPR, 66.7%) of preoperative chemotherapy with albumin-bound paclitaxel plus cisplatin and capecitabine in LA-ESCC.^20^ Our previous study found that nab-paclitaxel plus cisplatin could be efficacious and safe as drug therapy when combined with radiotherapy as conventional treatment for unresectable LA-ESCC and pCR was satisfactory up to 47.6%.^21^ Besides, immunotherapy has also demonstrated favorable antitumor effects in this patient population, with widespread use spanning from first-line to neoadjuvant treatments.^14,22^ Milestone KEYNOTE-590 trial reported better survival outcomes (OS, 12.4 vs. 9.8 months; progression-free survival, 6.3 vs. 5.8 months) of pembrolizumab combined with platinum-based chemotherapy (cisplatin/5-FU) as the first-line therapy for advanced ESCC compared with chemotherapy alone.^14^ Despite the exciting results from these studies, immunotherapy alone or in combination with chemotherapy may not be the optimal choice for each patient.^23,24^ A meta-analysis pooled first- and second-line trials for advanced ESCC showed no significant OS benefits with chemoimmunotherapy over chemotherapy in the patient subpopulation with a tumor proportion score below 1% (hazard ratio [HR], 0.91; 95% CI, 0.74–1.12; *p* = .38).^24^ Generally, switching to other medications or radiotherapy-based regimens was available for these patients who did not respond effectively to immunotherapy.

There is no established standard treatment for LA-ESCC patients who failed to first-line treatments. Current second-line therapies primarily focus on immunotherapy-based regimens for advanced ESCC patients.^25–27^ Our S-1-Nimo regimen achieved a numerically higher OS (13.4 vs. 7.2/10.9 months,^25,26^ indicating its potential as an appropriate approach to guide the clinical second-line treatment of LA-ESCC. The addition of radiotherapy might mainly contribute to favorable treatment outcomes. However, for patients with no prior anticancer treatment, our previous study, evaluating survival in inoperable ESCC patients after definitive (chemo)radiotherapy, demonstrated a median OS of 22.1–22.9 months for those in TNM stage III-IV,^28^ which was significantly better than that (13.4 months) in this study. Moreover, Sehhoon Park et al. reported that durvalumab plus tremelimumab and definitive CCRT had promising efficacy in LA-ESCC patients, especially with high PD-L1 expression, which showed a superior 24-month OS rate compared with ours (85.7% vs. 31.2%).^29^ Overall, patients with poor response to chemotherapy/chemoimmunotherapy in our study exhibited lower sensitivity to radiation and had a prognosis inferior to those who initially received combined chemo(immuno)therapy and radiotherapy.

Generally, patients experienced reduced tolerance, including bone marrow suppression and worsening performance status, after the failure of prior chemotherapy or chemoimmunotherapy.^16^ Thus, we used low-toxicity S-1 chemotherapy in this combination regimen for enhanced tolerance of patients; besides, S-1 could also exert antitumor effects by the inhibition of the deoxyribonucleic acid synthesis and favor radio-sensitization by preventing the degradation of 5-FU.^30,31^ Our previous randomized trial also showed the favorable safety and efficacy of S-1-based CCRT in LA-ESCC patients aged ≥70 years.^32^ Meanwhile, the addition of nimotuzumab may also be highlighted as a potential reason for promising efficacy data because the preclinical study showed that nimotuzumab could upregulate IGFBP-3 to promote radio-sensitivity and subsequently translate into antitumor efficacy in LA-ESCC.^33^ A randomized phase II NICE trial reported that compared to CCRT alone, CCRT plus nimotuzumab increased endoscopic pCR (cEPCR; 62.3% vs. 37.0%, *p* = .02) rate and was safe in locally advanced esophageal cancer. Besides, our previous work in a real-world setting also suggested an impressive OS (48.4 months) and manageable safety with CCRT plus nimotuzumab for elderly patients with LA-ESCC.^34^ Further, the interim analysis of a randomized, double-blinded, placebo-controlled phase III trial demonstrated that nimotuzumab plus CCRT increased ORR (93.8% vs. 72.0%, *p* < .001) and a favorable safety profile (grade 3–5 adverse drug reactions were 11.1% vs. 10.9%; *p* > .05).^18^ Overall, given these effectiveness and low-toxicity data characterizing S-1 and nimotuzumab, the combination of S-1-Nimo-based CCRT may be feasible for cancer treatment, as demonstrated in a case report of pancreatic cancer using this combined regimen.

As demonstrated in the RICE-Retro real-world study evaluating the feasibility of conversion surgery after chemotherapy or chemoimmunotherapy in initially unresectable LA-ESCC, EFS was significantly higher in patients with conversion surgery than those without the surgery, and pCR rate was 22.4% after chemoimmunotherapy.^35^ Our previous study has confirmed that patients who are converted to surgery after chemoradiotherapy survive well (OS rates at 1-year/2-year were 64.3%,73.2% and EFS rates were 72.4%, 68.8% respectively) and the pCR is nearly 47.6%.^21^ Consistently, subgroup analysis data presented here that patients who received radical surgery have obviously prolonged EFS (*p* = .0117) and OS (*p* = .0205) than those without surgery. In addition, numerically higher pCR (33.3% vs. 22.4%) and R0 resection rate (100.0% vs. 94%) were observed compared to those in the RICE-Retro study despite patients in our study was with poor response to initial chemotherapy/chemoimmunotherapy. However, cross-trial comparisons of surgical outcomes should be interpreted cautiously due to the relatively low surgery conversion rate of 18.8% in the present study. We also need to explore more effective radiotherapy concurrent drugs, proper radiation dose, or target delineation, in order to improve radiotherapy sensitivity and surgical conversion rate for those refractory to chemoimmunotherapy patients.

Equally important is the consideration of risk and management in terms of treatment-related toxicity. The preoperative addition of S-1-Nimo-based CCRT was well-tolerated even after several cycles of chemo (immuno) therapy administered. Safety profiles were consistent with previously reported,^19^ and there was no new safety signal. Gastrointestinal and hematological toxicities and radiation-induced esophagitis were commonly observed in our findings, which was in line with those reported previously.^17,18,36^ Besides, the rate of ≥grade 3 radiation esophagitis was numerically lower in our study than in the prior studies.^36^ Although the overall incidence of radiation esophagitis was high in this study (76.6%), the incidence of grade 3 or higher was only 4.7%. This may be related to the fact that we changed the standard concurrent chemotherapy regimen of paclitaxel plus platinum to S-1 plus nimotuzumab. At the same time, we pay great attention to the treatment of radiation esophageal cancer, using traditional Chinese medicine (Kangfuxin, a new rehabilitation liquid), oral nutrition support for patients with nutritional risk as soon as possible, gargling to maintain oral hygiene, and giving painkillers, etc., which have achieved good results, significantly reduced the incidence of grade 3 and above radiation esophagitis, alleviated the symptoms of patients, and improved the quality of life. Of note, enrolled patients demonstrated high compliance with S-1-Nimo treatment, reflected in the fact that 95.3% and 87.5% of patients respectively completed radiotherapy and chemotherapy. In addition, there were no severe or life-threatening postopertive complications occurring in our study. Overall, the S-1-Nimo regimen exhibited manageable safety profiles, especially considering the fragile condition and myelosuppressive status of the patients after the failure of neoadjuvant therapy.

There were some limitations in our study. First, this real-world study from a single institution in China might lead to potential selection bias and the potential confounding factors resulting in inaccuracies for our results. Second, the relatively limited sample size (64 patients lacking of prospective sample size calculations) may affect the statistical significance reducing the reliability, the uneven number of patients treated in the two groups and subsequent treatment methods were not strictly the same, affecting the generalizability of the results. These questions can be addressed in a multicenter/prospective randomized controlled trial in the near future to confirm our findings. Third, given that the association between PD-L1 expression and the survival of patients is currently inconclusive, the PD-L1 status was thus not assessed in our study.

## Conclusions

For LA-ESCC patients who failed to neoadjuvant chemo(immuno)therapy, the S-1-Nimo-based CCRT was a potentially feasible treatment strategy, with reasonable efficacy and promising safety. A large-scale randomized trial for further evaluation of this regimen and predictive biomarkers for survival outcomes is warranted.

## Supplementary Material

Supplementary table S1.docx

## Data Availability

The datasets supporting the results of the present study can be obtained from the corresponding author upon reasonable request.
