# Supplementary material for: S-1-based concurrent chemoradiotherapy plus nimotuzumab in patients with locally advanced esophageal squamous cell carcinoma who failed neoadjuvant therapy: a real-world prospective study
Source: Cancer Biol Ther. 2024 Oct 27;25(1):2417464. doi: 10.1080/15384047.2024.2417464 (PMC11520572; doi:10.1080/15384047.2024.2417464)
Supplement: Supplementary table S1.docx [file KCBT_A_2417464_SM8849.docx]

# Supplementary Table S1. Subgroup analysis of the relation between clinical factors and event-free survival/overall survival.

| Characteristics | No. of patients | Median EFS, months (95% CI) | *p*-value | Median OS, months (95% CI) | *p*-value |
| --- | --- | --- | --- | --- | --- |
| Age |  |  | 0.5560 |  | 0.4670 |
| ≥70 | 11 (17.2%) | 14.9 (3.6-18.7) |  | 17.4 (3.6-NE) |  |
| <70 | 53 (82.8%) | 8.9 (6.7-10.9) |  | 12.2 (10.0-17.5) |  |
| Sex |  |  | 0.2761 |  | 0.1743 |
| Male | 59 (92.2%) | 9.4 (6.9-14.0) |  | 12.2 (10.1-17.4) |  |
| Female | 5 (7.8%) | 14.9 (6.3-NE) |  | NR (10.6-NE) |  |
| Smoking history |  |  | 0.3863 |  | 0.4303 |
| Yes | 55 (85.9%) | 8.9 (6.7-12.7) |  | 12.2 (9.7-17.5) |  |
| No | 9 (14.1%) | 14.9 (4.9-NE) |  | 17.4 (4.9-NE) |  |
| ECOG PS |  |  |  |  |  |
| 0-1 | 59 (92.2%) | 9.6 (6.9-14.8) | 0.4710 | 15.7 (10.4-19.2) | 0.1060 |
| 2 | 5 (7.8%) | 10.1 (5.0-NE) |  | 10.1 (5.0-NE) |  |
| Alcohol history |  |  | 0.1449 |  | 0.1454 |
| Yes | 52 (81.3%) | 8.3 (6.4-14.0) |  | 12.2 (9.7-17.4) |  |
| No | 12 (18.7%) | 13.8 (8.9-NE) |  | NR (9.4-NE) |  |
| BMI |  |  |  |  |  |
| Thin (BMI under 18.5) | 9 (14.1%) | 10.8 (5.0-NE) | 0.9440 | 14.4 (5.0-NE) | 0.9946 |
| Normal (BMI 18.5-25.0) | 38 (59.4%) | 8.7 (6.4-18.7) |  | 10.9 (9.7-17.5) |  |
| Overweight (BMI greater than 25.0 and under 30.0) | 15 (23.4%) | 9.6 (1.9-14.9) |  | 15.7 (6.0-24.9) |  |
| Obese (BMI greater than 30.0) | 2 (3.1%) | NR (4.9-NE) |  | NR (4.9-NE) |  |
| TNM stage |  |  |  |  |  |
| III | 14 (21.9%) | 9.1 (4.6-18.7) | 0.9329 | 10.6 (6.9-NE) | 0.9774 |
| IVA | 31 (48.4%) | 10.8 (6.6-14.8) |  | 14.4 (10.1-17.5) |  |
| IVB | 19 (29.7%) | 9.6 (5.8-NE) |  | 13.4 (8.9-24.4) |  |
| Cycle of nimotuzumab |  |  | 0.6812 |  | 0.5906 |
| 1-4 | 27 (42.2%) | 9.4 (6.4-12.7) |  | 12.2 (9.4-16.3) |  |
| 5-6 | 37 (57.8%) | 10.3 (6.6-15.7) |  | 15.7 (10.1-22.2) |  |
| GTV |  |  |  |  |  |
| >41 | 32 (52.5%) | 6.8 (4.6-9.6) | <0.0001 | 10.1 (6.9-12.2) | <0.0001 |
| ≤41 | 31 (49.2%) | 15.7 (10.8-NE) |  | 24.4 (14.4-NE) |  |
| Anti-PD-1 at neoadjuvant stage |  |  |  |  |  |
| Yes | 43 (67.2%) | 10.8 (6.9-14.8) | 0.1933 | 14.4 (10.1-24.4) | 0.4006 |
| No | 21 (32.8%) | 8.7 (4.9-12.7) |  | 12.2 (9.4-16.3) |  |
| Tumor length |  |  | 0.0100 |  |  |
| >5 cm | 38 (59.4%) | 7.1 (5.0-8.9) |  | 10.4 (7.4-15.7) | 0.0060 |
| ≤5 cm | 26 (40.6%) | 14.8 (10.4-NE) |  | 22.2 (12.2-NE) |  |
| Surgery |  |  |  |  |  |
| No | 52 (81.3%) | 8.7 (6.4-10.8) | 0.0117 | 10.6 (9.4-16.2) | 0.0205 |
| Yes | 12 (18.8%) | NR (6.6-NE) |  | 24.9 (10.9-NE) |  |
| Cycle of neoadjuvant therapy |  |  |  |  |  |
| <4 | 36 (56.3%) | 9.8 (6.6-14.6) | 0.6060 | 12.2 (8.8-24.4) | 0.8950 |
| ≥4 | 28 (43.8%) | 8.7 (6.3-15.7) |  | 13.4 (10.4-19.2) |  |
| Best response at neoadjuvant stage |  |  |  |  |  |
| PR | 33 (51.6%) | 12.7 (8.7-14.8) | 0.8191 | 15.7 (10.3-22.2) | 0.7954 |
| SD | 22 (34.4%) | 7.7 (4.5-15.7) |  | 9.5 (6.0-24.9) |  |
| PD | 9 (14.1%) | 6.7 (2.4-NE) |  | 12.2 (10.1-NE) |  |

**Abbreviations:** PGTV, planning gross tumor volume; PTV, planning target volume; GTV, gross target volume; CI, confidence interval; PD-1, programmed cell death protein 1; OS, overall survival; No., number; NE, not estimable; NR, not reached; EFS, event-free survival; BMI, body mass index; ECOG PS, Eastern Cooperative Oncology Group Performance Status; TNM, tumor, node, metastases; PR, partial response; SD, stable disease; PD, progressive disease.
